# Mapping the hinterland: Data issues in open science

**DOI:** 10.1177/0963662514530374

**Published:** 2016-01

**Authors:** Ann Grand, Clare Wilkinson, Karen Bultitude, Alan F. T. Winfield

**Affiliations:** University of the West of England, UK; University of the West of England, UK; University College London, UK; University of the West of England, UK

**Keywords:** open science, public engagement, science communication

## Abstract

Open science is a practice in which the scientific process is shared completely and in real time. It offers the potential to support information flow, collaboration and dialogue among professional and non-professional participants. Using semi-structured interviews and case studies, this research investigated the relationship between open science and public engagement. This article concentrates on three particular areas of concern that emerged: first, how to effectively contextualise and narrate information to render it accessible, as opposed to simply available; second, concerns about data quantity and quality; and third, concerns about the skills required for effective contextualisation, mapping and interpretation of information.

## 1. Context

The Internet has completely changed the relationship between provider and user, producer and consumer, expert and novice (Bruns, 2009), as the boundaries between public and private, accessible and closed, have become more porous (Trench, 2008a). Such direct, unmediated access could allow a far wider range of participants – both professionals and non-professionals – to engage with research in an unprecedentedly complete way. Where once only the refined, polished outputs of research appeared in public, leaving its hinterland to be explored by knowledgeable and qualified residents, those uncharted territories can now be open to inexperienced travellers.

The roots of open science can arguably be traced to the formation of the first scientific societies in the seventeenth century. These societies represented a revolutionary organisational change, from a culture based on secrecy and patronage to one of professional reputation and autonomy (David, 2008; Nielsen, 2012; Schroeder, 2007). However, the demand for open access to information has become louder in the twenty-first century, as part of a cultural trend in which ‘open’ prefixes an ever-wider range of endeavours: government, culture, archives, research, knowledge, source, data, democracy, journalism and more. This demand is accompanied by a trend towards community collaboration, production and co-operation, with some participants ‘working for nothing and beating the pros at their own game’ (Grossman, 2006). Academic research has not escaped this trend. However, greater openness may require more than a change in practice; it may involve an evolution of philosophy, as Burton (2009) suggests,

The ‘Open Scholar’, as I’m defining this person, is not simply someone who agrees to allow free access and reuse of his or her traditional scholarly articles and books; no, the Open Scholar is someone who makes their intellectual projects and processes digitally visible and who invites and encourages ongoing criticism of their work and secondary uses of any or all parts of it – at any stage of its development.

Practising open science philosophically commits researchers to revealing and sharing the entirety of their data, methodologies, results and models (Nielsen, 2009). ‘Entirety’ can be diverse: funding applications, paper drafts, meeting minutes, day-to-day methods, experimental data and final publications; therefore, open projects have the potential to support public engagement with research throughout the process.

The strategies of public engagement are highly dependent on the time, culture and attitudes of the societies in which they are practised: both discourse and understanding have evolved considerably and appeared under many labels (Bauer, 2009; Bauer et al., 2007; Burns et al., 2003). The trend – most readily seen in the United Kingdom and the United States – from expert homily to mutual engagement, from one-way transmission to multi-way dialogue has, since the mid-1980s, increasingly allowed ‘people with varied backgrounds and scientific expertise [to] articulate and contribute their perspectives, ideas, knowledge, and values in response to scientific questions or science-related controversies’ (McCallie et al., 2009: 12). However, some researchers have asserted that this move is neither complete nor irreversible (Trench, 2008b) and that moments of deficit may be found in the midst of apparent dialogue (Davies, 2009; Wilkinson et al., 2011).

Surveys of public attitudes to science (European Commission, 2010; Ipsos Market & Opinion Research International (MORI), 2011; National Science Foundation, 2010) suggest that people are willing to engage in dialogue with scientists, if opportunities exist. Although some researchers are unconvinced that the public has much to contribute to the analysis of results (Staley, 2009), others suggest that increasing public involvement can enhance the process, either by reflecting alternative perspectives or by contributing to the accessibility of research for particular user groups (Bell et al., 2008; FoldIt, n.d.; Powell and Colin, 2008). The ‘growing number of scientist-driven public research projects’ (Bonney et al., 2009: 15) – often labelled ‘Citizen Science’ – suggests people are willing to contribute to research, where they have sufficient expertise, skills and time (Blackman and Benson, 2010; Raddick et al., 2010). However, Citizen Science is not necessarily open, as ‘many citizen science projects share data, but may not make the full research process publicly viewable for comment and discussion’ (Wiggins and Crowston, 2011: 2).

Open science offers both new modes for communication (De Roure et al., 2008) and new routes for public engagement with science (National Science Foundation, 2010). People can follow projects in which they are interested, search for information, and access data, publications and outputs. Open science could thus support public participation in research, enabling the collaborative design and creation of research projects, the co-operative collection and production of information, or the collective re-purposing of existing information. However, the question of how these new routes are mapped to support such wider participation remains to be answered.

To continue the analogy, when investigating new territory, it is vital to know from where one starts. Researchers are not now necessarily found in their traditional lairs in the physical buildings of universities or industries (Hess, 2011) but may be working in non-governmental organisations, charities or private homes. An unofficial or emergent counter-public exists (Hess, 2011), which uses alternative pathways, can arise from any social arena, be part of a community organisation (such as the ‘civic scientists’ (PLOTS, n.d.) involved in community-focussed research), belong to no organisation or move among all these situations. The Internet supports the creation of dynamic, self-organised, shifting networks of individuals, and therefore the emergence of new counter-publics, by offering a space to:

unleash the diverse creativity of academic researchers, journalists, software geeks and mappers, who are often better equipped, and more agile than governments and international agencies, to present data online in timely, informative and compelling ways. (*Nature*, 2011: 135)

Opening research to the wider public has always raised issues. A criticism of public engagement that can be extended to open science is that it adds to researchers’ burdens and takes time from ‘real work’. A further issue revolves around how information is offered in public: to be truly useful, information must be presented in ‘high-quality, user-friendly forms’ (*Nature*, 2011: 135). Selection and translation into new forms inevitably reduce the circumstantial context that enables readers to accurately reconstruct an experimental scene and judge to what extent things have ‘been done and done in the way claimed’ (Shapin and Schaffer, 1985: 60). To extend the metaphor, since public participants are almost certain to be located ‘beyond the borders of the scientific community’ (Suleski and Ibaraki, 2010: 112), they will need accurate maps to help them navigate its interior, find the firm ground and avoid treacherous bogs.

The findings described in this article arise from PhD research that investigated the hypothesis that open science could support public engagement with science. Several challenges for practising open science emerged, of which this article focusses on three: how research can be contextualised, narrated and organised in ways that make sense beyond the research community; how to deal with issues of data quality and quantity; and the skills required of participants, both professional researchers and members of the public.

## 2. Methods

These findings derive from semi-structured interviews exploring the views of various groups about the implications and potential for public engagement with science of open science’s principles, methods and values, and case studies exploring practical implementations of open science. The protocol was approved through the University of the West of England’s research governance system.

The researcher used a grounded theory approach, suited to the study of phenomena in complex fields, where a combination of methodologies must be integrated (Charmaz, 2006). This ability to cope with complexity renders grounded theory suitable for new fields of study – as is open science – where theories and constructs are not yet well developed and existing data are limited (Creswell, 2007; Flick, 2007).

A total of 30 semi-structured interviews, lasting approximately 45 minutes, was conducted with 13 members of the public, 12 professional and amateur researchers in various fields and 5 professional and amateur public engagement practitioners. The interviews (except 4 conducted as a series of emails) were conducted verbally, either in person or by telephone, recorded and transcribed.

The interviews were conducted in two phases: a series of four pilot interviews and then the main series. The interview data analysis was emergent and inductive, with coding categories developed through analysis. As each new interview was analysed, the text selected was compared with previously coded selections until no new insights or properties were revealed (data saturation). This iteration continued throughout the 20 months of active research. Constant comparative analysis is one of the interpretive strategies that addresses perceived problems of the grounded theory approach and enhances its effectiveness as a methodology (Denzin and Lincoln, 1994). To test reliability, a randomly chosen selection of interviews was re-coded (using the same coding frame) by a second researcher, unconnected with the project, giving an agreement level of 80%.

The professional and amateur researchers and practitioners were identified through a combination of snowball, convenience and self-selective sampling, and direct identification from Internet and literature/media searching. Using more than one approach was necessary to ensure – as far as possible – that no group of potential interviewees was excluded from discovery. Such a research design, in which participants are iteratively selected to best develop the evolving theory, seeks to obviate the biases that can arise from these sampling methods, in addition to locating individuals in a scattered community (Creswell, 2007).

The first phase involved people working in fields related to this research. They comprised a professional scientist who practised open science, a researcher in public engagement, a member of the public who voluntarily organised public engagement events and an amateur scientist. The importance of involving an ‘interested party’ – a member of the open science community – in this phase lay in the fact that the philosophy of open science was in an embryonic, emergent stage, with its concepts and strategies still cohering; this conversation allowed the researcher to develop a more subtle understanding at an early stage. The analysis of the results of these interviews supported both the development of the interview structure and the identification of either appropriate future interviewees or areas of interest where interviewees were needed.

Members of the public were recruited through an emailed appeal to audience members of the UK café scientifique network.^1^ This route was chosen as it enabled the researcher to reach an audience spread throughout the United Kingdom, whose members were likely (by virtue of attending a café scientifique) to be interested in science but not necessarily professional scientists. However, using this route did mean that the pool of respondents was circumscribed and unlikely (though not impossible) to contain people completely uninterested in science. The appeal specifically asked for respondents who were not professional scientists; a small number responded but were not interviewed.

A semi-structured interview, with its use of flexible, open-ended questions, allows rich data to emerge. Nevertheless, to allow reliable data comparison, the nucleus of the questions remained largely consistent (Strauss and Corbin, 1990). For professional researchers and practitioners, the questions covered their experience of public engagement with science; assessments of barriers to engagement; understanding, perceptions and experiences of open science; and views on the potential relationships of open science and public engagement. Questions for members of the public and amateur researchers additionally addressed issues of access to and availability of information, public engagement and expertise. A departure from convention was that, in the spirit of openness, interviewees chose whether to be named or anonymous. Interviewees were classified into four descriptive categories, based on interviewees’ free-form self-descriptions: researcher, public engagement practitioner, amateur scientist or member of the public.

The case studies were selected using a method based on Denzin and Lincoln’s (1994) approach of developing a typography and using it to set up a descriptive decision matrix that thoroughly describes the phenomenon under study. The matrix was used to assess 20 active (or in development) projects against three groups of criteria. Most criteria were derived from literature, apart from two (specific to public engagement through open science) that emerged from early interview data. One group related to evidence of public engagement in the projects (e.g. participation by both experts and non-experts; Poliakoff and Webb, 2007); the second concerned the extent to which projects practised – or promised to practise – open science (e.g. were raw data available; Science Commons, n.d.); and the third covered elements that would tend to support public engagement through openness (e.g. were public contributions facilitated; McCallie et al., 2009). The three projects that emerged from the matrix with the highest scores were pursued as case studies. Project A was a multiple-partner, university-based, robotics research project; Project B was an archaeology project with both academic and industry partners; and Project C was a mostly privately-funded single-site engineering project.

The basic documentary evidence for the case studies came from the projects’ websites, reviewed approximately every 2 months to offer a longitudinal view of their development. The websites were analysed to establish parameters such as types and quantity of data available, for example, experimental data, project documents and publications, news and background information, numbers and authorship of postings and comments on project blogs. By invitation, meetings and events of projects A and C were observed and field notes, including personal reflections, were recorded (Gillham, 2010). Members of Projects B and C were interviewed at the start and end of the research. (It was not possible to interview members of Project A.) Although returning to interviewees might be considered as introducing an element of bias, in that they were more aware of the aims and objectives of the research, it allowed the researcher to compare responses over time and interviewees to reflect on their practice and developments within their projects. These interviews were captured and analysed using the same grounded theory approach as the main interview series.

## 3. Findings

As noted earlier, at its fullest, open science involves making ‘data, scientific opinions, questions, ideas, folk knowledge, workflows and everything else – available online and as it happens’ (Nielsen, 2009: 32). This is quite an extreme stance: researchers acknowledged that, in practice, openness was a continuum on which they occupied different positions:

It ranges from simply making regular articles free to the public (Open Access) to sharing every detail of laboratory work in progress (Open Notebook Science^2^) (Bradley, researcher)

Although some researchers are comfortable ‘sharing every detail’, others judge to what level of detail they are open. However, sharing means more than simply making information available:

‘Available’ for me, is about … it needs to mean something to the person that’s accessing it. It’s only ‘available’ if it means something; it’s not ‘available’ if it’s just there but means nothing or there’s no map to navigate through it in some way or no support to find your way through. (Anonymous 1, researcher)

### Mapping the hinterland

Several interviewees suggested that to be useful to audiences beyond immediate colleagues, outputs needed to be annotated and contextualised:

You need to know the hinterland of the data, the context in which the data can be set. (Horton, amateur scientist)

However, mapping data so as to give a comprehensible sense of context was noted as potentially difficult, given the nature of its basic form:

As regards presenting that out to the public, that’s going to be hard. It’s going to be a lot of machine-processed digital data – hardly sexy! (Beck, researcher)

If you look at the raw data that comes from a satellite about … sea level height … it’s huge numbers of 1s and 0s. You cannot do anything with it. It needs to be processed, it needs to be dealt with. (Murcott, practitioner)

As Murcott continued, this very rawness makes contextualisation ‘utterly, utterly, essential’. Researchers already spend time creating narratives for their work, refining the information according to the milieu in which it will appear: seminars and papers for fellow specialists, talks and journalism for the wider public, textbooks for teaching and so on. This demand for – or wish to supply – contextualisation can be seen as simply more work or as removing the focus from ‘real work’. These demands on researchers’ time seem magnified by the lens of social media: Crotty (2010) suggests ‘every second spent blogging, chatting on FriendFeed, or leaving comments on a *PLoS* paper is a second taken away from other activities [that] have direct rewards towards advancement’. Neylon and Hendy expressed similar concerns:

It’s difficult to persuade people to take time because you don’t see how it [open practice] can get built into your standard work pattern. (Neylon, researcher)

… if I’m in my office, I feel guilty blogging. So a lot of it’s done in my personal time. That’s partly because there’s a lot of time demands, doing science … even if I could say it was a core part of my job, I’d still find it hard, finding time in my working life to put time into that. (Hendy, researcher)

The need for time was reflected in the case studies, which, in differing ways, had experienced problems in making information and resources available. In the blogs of projects A and B (C had no blog), the contributions of project members were uneven and neither posting nor comments were regular or rich. At the time of study, Project B had 16 posts, written over approximately 12 months, by 4 (of 5) project members. These posts had generated 16 comments, of which five were written by project members. Project A had 31 posts, written over approximately 18 months, by 4 (of 12) project members; all the comments were from project members. This low level of involvement is not in itself remarkable on interactive websites: ‘in most online communities, 90% of users are “lurkers” who never contribute, 9% of users contribute a little, and 1% of users account for almost all the action’ (Nielsen, 2006).

However, other considerations affect why certain participants may be reluctant to contribute. Different disciplinary ethos can lead to unequal approaches to making information available, especially visible in multi-disciplinary projects:

So far my reasons for ‘not yet’ [posting interview data] are bound up with the research disciplines in which I work – sociology and health sciences. (Project A blog post; researcher)

Confidentiality is an important concern for researchers whose work involves people, as the post above implies. While some medical archives have high deposition rates, researchers have expressed concerns about retaining the confidentiality of patients’ information (Nelson, 2009). Just as members of different professional groupings may have differing attitudes towards open practice, members of different public communities may well have differing expectations about the uses and opportunities of openness. The shift of language from one ‘public’ to multiple ‘publics’ reflects the interpretation that while every person in a society is a member of the public, societies contain fluid sub-groups that form, re-form and overlap, depending on their interests, backgrounds, experiences and preoccupations (Braun and Schultz, 2010; Burns et al., 2003; McCallie et al., 2009). For example, patients themselves are beginning to overturn deep-seated notions of personal privacy, as shown by websites where people living with a variety of medical conditions share their experiences and in the course of participation identify themselves (PatientsLikeMe, 2012).

The members of the public and amateur scientists interviewed for this project suggested open practice opened three key possibilities. First, it offered a means by which non-professionals could contribute to research:

In theory, my own papers would also be made available to a wider audience and in this way they could finally enter the main stream of scientific discourse. (Anonymous 3, amateur scientist)

Others noted that open practice could allow members of the public to contribute to projects, either through offering their time and skills or through their stimulating watchfulness:

to talk, to start contributing to things you feel you know something about or happen to be in the right place for. (Pepperdine, member of the public)

… the fact they’ve got all these amateur … amateur but interested people watching means they [scientists] might discover something they wouldn’t have spotted themselves. (Anonymous 7, member of the public)

Second, open practice could be a route to sustain dialogue and conversation among scientists and members of the public, particularly, as McKay suggests, about issues:

… conveying the results … particularly, I’d suggest, about the political and ethical implications of the issues science raises. To my mind, that’s the most important thing; people don’t necessarily need to know all the nitty-gritty details […] It’s got to be about distilling the implications of that, so that public policy can be steered in an informed way. (McKay, member of the public)

Third, interviewees felt open practice supported access to information:

If you want to get hold of the actual paper – follow it up – then there’s a problem sometimes. (Pepperdine, member of the public)

[information would] be easily accessible to all citizens – regardless of their social or economic status. It would also facilitate access to all aspects of science: both historical and current research. (Nason, amateur scientist)

However, other interviewees conceptualised something quite close to a ‘deficit view’, in which open practice was a mechanism for conveying information or explaining new work:

I imagine open science is making science open to non-scientists to understand and get interested in. (Marks, member of the public)

… explaining it to the general public, not only to the community that the research is in. (Cumming, member of the public)

Earlier, the potential of open science to reflect a complete record of the research process was noted; a record that could, for example, include elements such as data, methodologies and publications. Of the three case study projects, Project A had made raw data available; however, these came from only one research group. The presence or absence of information cannot wholly be laid at the door of constraints on researchers’ time. Project C offered six sets of graphics/design drawings and two case study project specification documents on its website; these resources remained unchanged for over a year. This project faced difficulties in implementing consistent curatorial practices across a wide range of creative individuals (both professionals and volunteers) with different working practices and different team dynamics:

… to be open, I think we needed to have a structured approach, we needed to have things in place that meant we were able to store, archive, curate data in ways that made it easy for that then to be accessible and open. (Project C member)

In Project C, the desire to allow the creative team to work unhindered, allied to a considerable time-lag between the project starting and efforts to begin curating the data, meant that information had been produced and stored idiosyncratically. Attempts to impose a structure that would allow the data to be gathered, organised and stored in a way that made it accessible to its target audiences proved insufficient, in that the amount of time required – and the need – to interpret, mediate and categorise the data was greater than forecast. This highlights the necessity for project teams to plan and agree communication and information structures that will sustain openness from the beginning.

As Neylon noted, further difficulties arise from the many different sources where information might be found and the many different uses to which it might be put:

… My lab notebook is in some ways the bottom layer of the record. It’s almost the machine code kind of level: ‘this happened – that happened – this happened’. There’s often a tension in that record about actually putting reasons, rationale and analysis in at that level. It doesn’t seem to feel right; it doesn’t fit terribly well in the information framework as we have it. My strong suspicion is that we need some sort of layer on top of that. Maybe you need several layers of reporting, of analysis. (Neylon, researcher)

How and when information can or should be shared is becoming one of the more contentious issues in current science. As Neylon speculates, should it be raw data, at the machine level or should it be refined, normalised and accompanied by rationale and analysis? The economic value of making data available for re-use and re-purposing has been acknowledged (European Commission, 2011) but established systems for reward and recognition have yet to be adapted to acknowledge the evolution from the time-honoured but relatively closed process of ‘work, finish, publish’ (Hamilton, 2003) to a more open, dynamic process. The Royal Society (2012) explicitly acknowledged the increased demand from citizens, civic groups and non-governmental organisations for access to evidence that would enable them to scrutinise conclusions and participate effectively in research. Such demands ‘for openness and access to data are, like it or not, indicative of a transformation in the way science has to be conducted in the twenty-first century’ (Russell, 2010: 15).

### Quality and quantity

Non-professionals’ increased participation in research, whether as contributors, collaborators or co-creators, has brought questions of whether the quality of the information they provide is ‘good’ enough and whether this is an issue that needs to be addressed (Riesch and Potter, 2013). Horton – an amateur scientist – said,

… we do need to know about the quality of that data … some write-up about its quality assurance and how it was got […] It’s all very well saying ‘let’s just open the doors to the data’ – I just want it to be done responsibly […]. (Horton, amateur scientist)

Currently, there is no generally recognised method for measuring quality or assessing the trustworthiness of websites’ information. Various systems are used to develop and maintain the reputation of information and information-providers: collaborative filtering asks users to make assessments of trustworthiness, pooling judgements and experiences (Metzger, 2007); social reputation measures allow users to acquire increasing reputation if their responses are judged high-quality or proved accurate, or lose it if they are inaccurate (Clow and Makriyannis, 2011; MathOverflow, n.d.). Collaborative projects have used both pre-submission testing and training and post-submission (error-identification and data cleaning) measures to ensure community-generated data are acceptable (Galaxy Zoo, 2010; Worthington et al., 2011). However, the anonymity and unregulated nature of most open systems means that traditional methods for assigning trust – such as knowing that the source of the information is controlled or that it has been scrutinised by peer reviewers, professional editors or similar filterers (Keen, 2008) – are not available. This, and the sheer quantity of information available on the web, can make it difficult for high-quality, rigorously written sites to differentiate themselves from sites of lower quality and less thorough production.

This question was being considered by one of the case study projects, Project B. Community groups participating in this project wanted their work to be useful and viewed as good quality:

Both community groups and commercial practitioners feel constrained in what they can achieve in terms of data quality. […] It is anticipated by this audience that [the project] could help them acquire better data by producing clearer guidance as to when they should survey [and] create a set of protocols to establish better practice for obtaining high quality data. (Project B website, summary of community workshop)

In response, the project team planned to create an ‘open methods store’ in which developing methodologies could be shared by all participants:

We have an open methods store, which we haven’t started populating yet but we hope we’ll get out really soon. It’s probably going to start as a wiki base, where you deposit your method but being a wiki-based thing, the nice thing is we can start to discuss our methods and how they change, so we’re then collecting the history of the development of the science. (Project B member)

A further issue is that much of the information made available by open science flows through digital and social media channels. This, as Anonymous 4 suggested, could affect its perceived, if not its actual, quality:

… do you think using Twitter and Facebook devalues the science? I just don’t have a high regard for them; I think if I saw science coming out of them I’d almost think it was pseudo-science, a bit trashy really, not well thought through or considered. I might take that view without even reading it or looking at it. (Anonymous 4, member of the public)

In terms of quantity, the difficulties for consumers in managing large quantities of raw information were widely noted by interviewees. Horton, as an amateur scientist, was able to look at the problem both as a consumer and a producer. As a producer, he acknowledged that he faced problems of quantity:

I have got an awful lot of data, because this is now an automated system that collects just about everything every ten minutes. There’s an awful lot that I don’t make available on the web because it’s just too much for me to manage. (Horton, amateur scientist)

The problems of quantity can be the physical problems of large files and unwieldy datasets or of multiple hardware and software formats:

I don’t know what kind of raw data I would be able to use and I imagine there would be an awful lot of it. What do I do with that? In what form is the data going to be accessible to the public? Is it just going to be a photocopy of lab books? Is it going to be the scientists’ summary of the data …? (Foster, member of the public)

For another amateur, the problem was not just format or rawness; he noted the need for organisation and filtering:

It’s not enough just to upload papers and place them on the web. I would like to see some sort of initial assessment or filtering process. Papers that purport to have solved the Riemann Hypothesis using only simple arithmetic, or prove the existence of Bigfoot, or some such nonsense like that should not be allowed (Anonymous 3, amateur scientist)

Although Anonymous 3 made no suggestion as to who might do any filtering, the implication is that such assessment must be performed by someone who has the skills needed to ‘vouch for the reliability or credibility of the content’ (Keen, 2008: 65) and appraise the information. Summarising and filtering undoubtedly conflict with the philosophy of access to complete datasets. Above, Horton noted that he found it difficult to make all his data available; as a consumer, his view was different:

You don’t just give a sub-set, you give the whole lot. That’s what I would expect, what I would want. (Horton, amateur scientist)

There is tension between giving as much data out as possible and giving it in a form in which it is meaningful and usable. This is analogous with the tensions inherent in ‘citizen journalism’, which has, since the late 1990s, revolutionised the ways in which news is received, gathered, produced and disseminated. Members of the public can be both information-providers and information-seekers, with access to precisely the same sources as professional news reporters (Trench and Quinn, 2003). However, despite such changes, ‘professional news organisations still retain a very privileged place in framing and shaping the news agenda’ (Holliman, 2011: 2). The expertise of researchers is likely to mean that they will be awarded similar privileges, while also being afforded access to the expertise of public collaborators and contributors.

### Learning new skills or re-working old ones?

Much of the activity that renders science ‘open’ can be viewed as an evolution of older practices: the research notebook becomes a blog, the community becomes virtual, collaboration on papers is mediated via cloud storage and data are automatically collected and shared via a wiki. Despite being evolutionary, learning to use new tools nevertheless requires participants to acquire new techniques. Where the techniques required go beyond modifying existing skills, competencies other than practical skills may be required. For example, the development of narrative places considerable demands on creators to develop skills that may never before have been in their repertoire. Baram-Tsabari and Lewenstein (2013) noted, for example, that very few scientists use narrative in their communications, which tend to emphasise scientific content over discussion of the nature of the work.

Participating in open research seems likely to require both professional and non-professional participants to develop new skills, either in using new tools, such as social media, or in practising new processes, such as creating high-quality analyses. While some will be comfortable, others will struggle:

We all had the realisation that what we were asking people to do was unrealistic and we had people who not only struggled with online systems, or perhaps weren’t as digitally literate as we assumed they would be but we also had people who had no strategies for managing their own stuff, let alone for sharing it or packaging it or describing it with xml or anything else. (Millard, researcher)

While not a new issue, language remains problematic; as Guinamard noted, difficulties can be born of a lack of shared language:

[an] original paper would probably be too technical for me. If it were written in English – everyday English – I might read it! (Guinamard, member of the public)

Language use has long been a challenge in science communication. How to ‘forge a stable plane between scientific and nonscientific speech’ (Montgomery, 1989: 52) and to make scientific texts penetrable to non-specialists (Myers, 1997) has been under discussion since the seventeenth century, when, within the space of a few months, the Royal Society’s *Philosophical Transactions* was published in the United Kingdom and the *Journal des sçavans* in France. While the difficulties of language have largely been framed by the requirements of communication between researchers and members of the public, as Neylon noted, researchers working in different fields can experience similar difficulties:

It’s not ‘here are these people with their pointy heads who are somehow different to other people’. It’s that ‘here is a bunch of people with specific domain knowledge that speak specific sets of dialects and can converse with each other’. (Neylon, researcher)

Successful communication involves being aware of the needs of the audience; being conscious of those needs could, as Foster described, induce clearer communication:

I can also imagine, for example, reading the notes and not being able to understand them fully because they’d just been written for … whoever … I understand my notes; who else cares? Whereas I think that it would breed a wider sense of awareness in what you’re doing if you were making your notes for … You’d be thinking, have I made this clear? Have I made this in a logical sequence? Have I ordered my notes properly or are they all random? (Foster, member of the public)

Not only researchers may be required to develop new skills, to gain access to, interpret and understand the structures of the digital ‘collaboratory’ (Wulf, 1993), members of the public may also need new skills in enquiry and analysis. The greater availability of data offers both the opportunity to develop those skills and the material on which to practise them; as Murcott commented, the information could itself be a context in which users can develop skills in filtering and sifting layers of information:

Once you are immersed in the blogosphere, then you will start to develop those journalistic skills yourself. You will start to be able to say ‘this person here, is left-field, outlier, rarely brings anything other than random rants, whereas this person here is a provider of good-quality information and something I should be aware of’. (Murcott, practitioner)

These skills, and their expression through the medium of the Internet, matter ‘not least because by allowing people to participate and share, it also gives them a route to recognition’ (Leadbeater, 2009: 229), allowing the contributions of amateur scientists to be both valued and valuable.

The considerable growth in use of social media tools has brought open science practices more readily within the reach of both researchers and members of the public: writing blogs, commenting, micro-blogging, social citation software, video sharing, podcasting, and so on are increasingly commonplace. Rather than needing to develop skills de novo, both professional and non-professional participants are more likely to re-purpose existing skills and integrate them with traditional work and communication (Crotty, 2011).

## 4. Conclusion

Jasanoff (2006) wrote that while openness may be a ‘treasured attribute’ of science, to serve its purpose well, it must be ‘purposefully cultivated and judiciously deployed’ (p. 42). The capacity for open practice to support public engagement with science lies first in its capability to become embedded, allowing communication to arise from everyday activities and researchers to incorporate communication and dialogue within their work. Second, its mediation through Internet and web-based technologies means participants can not only contribute information but also share ideas, comment on and use information. Being a complete record, open science can reveal the complex workings of research. However, as Borgman (2003) suggested, making ‘digital laboratories useful to multiple audiences requires simple analytical structures, more common vocabulary and user interfaces that demand minimal domain knowledge’ (p. 165).

The research outlined in this article indicates three particular areas of concern regarding open science and its relationship with public engagement. First, contextualisation and narrative are key to supporting public engagement with research; offering open data in a form that is accessible, as opposed to simply available. Narratives and contextualisation will have to be created by information-providers, inevitably making demands on their time. However, demands on providers’ time may be justifiable as funders (Engineering and Physical Sciences Research Council (EPSRC), 2013; OpenAIRE, 2011; Wellcome Trust, n.d.) and governments (The Department for Business, Innovation & Skills (BIS), 2012; Holdren, 2013) make open access to the results of publicly-funded research mandatory. However, although the (UK-focussed) Finch Report concluded that the principle that publicly funded research should be openly accessible is ‘compelling and fundamentally unanswerable’ (Finch, 2012: 5), the case studies in this research showed that how or when information should be made open remains under discussion: ‘although many researchers have recognised that this shift is essential for projects to become genuinely collaborative, no one has reported finding it easy’ (Staley, 2009: 66).

Second are concerns about the quantity and quality of data produced from open collaborations. Producers are likely to find it difficult to store, archive and curate data in consistent and useful ways and consumers may find it hard to navigate a vast flow of data. Allied to this are concerns about the quality of data produced by non-professional participants and how its credibility and trustworthiness can be judged. In the digital environment, where ‘nearly anyone can be an author [and] authority is no longer a prerequisite for content provision’ (Metzger, 2007: 2078), conventional indicators such as authors’ institutional affiliation and the reputation of particular publications may not be well understood. To sustain open science’s potential to enhance public engagement, further attention must be paid to the development of mechanisms that support mutual respect, dialogue and collaboration.

Third are concerns about the skills required for effective contextualisation, mapping and interpretation of information. While researchers may see openness as demanding new skills, many are likely to be an evolution of older practices, as open science makes use of increasingly familiar Web 2.0 tools and techniques (Alexa, n.d.). Currently, relatively small numbers of researchers make use of such tools (Research Information Network, 2010); while they may be perfectly comfortable using them in private life, they have yet to transfer them to professional practice. For their part, information consumers may need new skills in gaining access, interpreting and understanding research. However, the greater availability of data may itself offer both the opportunity to develop those new skills and the material on which to practise them.

As open science becomes more widespread, professional and non-professional researchers will have to grapple with issues of how to share the ownership of research, with changing understandings of who constitutes a ‘researcher’ and with new kinds of hierarchies of roles and expertise: an area which offers rich potential for continued research.

## Notes

1. See www.cafescientifique.org. Café Scientifique is a largely voluntary network running (at the time of the research) in approximately 60 towns and cities in the United Kingdom and approximately 300 locations around the world. Typically, cafes meet once a month to hear a working scientist discuss their current work, with majority of the time spent in informal discussion between the speaker and the café participants.

2. In ‘Open Notebook’ research, researchers record their work using electronic laboratory notebooks, posting the contents on the Internet ‘for public scrutiny […] in as close to real time as possible’ (Stafford, 2010: S21).
